# Urinary Titin as a Non-Invasive Biomarker for Sarcopenia Sex Differences in Unresectable Digestive Malignancies: A Retrospective Cohort Study

**DOI:** 10.3390/ijms26146781

**Published:** 2025-07-15

**Authors:** Shiho Kaneko, Kazuaki Harada, Masatsugu Ohara, Shintaro Sawaguchi, Tatsuya Yokoyama, Koichi Ishida, Yasuyuki Kawamoto, Satoshi Yuki, Yoshito Komatsu, Naoya Sakamoto

**Affiliations:** 1 Division of Cancer Center, Hokkaido University Hospital, Sapporo 060-8648, Japan; kaneko.shiho.s8@elms.hokudai.ac.jp (S.K.);; 2 Department of Gastroenterology and Hepatology, Hokkaido University Hospital, Sapporo 060-8648, Japan

**Keywords:** urinary titin, sarcopenia, digestive malignancies, diagnostic marker, sex differences

## Abstract

The prognosis of sarcopenia is poor in cancer patients. Recently, urinary titin, a biomarker of muscle damage, has been suggested as a potential marker for sarcopenia. However, its utility in patients with unresectable digestive malignancies remains unclear. In addition, sex differences have been reported in the association between sarcopenia and urinary titin levels. This study aimed to evaluate urinary titin as a diagnostic marker for unresectable digestive malignancies, focusing on sex differences. This retrospective study enrolled 96 patients (58 males, 38 females; median age 70), and urinary titin was evaluated as a diagnostic biomarker in relation to clinical factors (e.g., age, Eastern Cooperative Oncology Group performance status [ECOG PS], albumin [Alb]) and muscle indicators (e.g., psoas muscle index [PMI], handgrip strength). In male patients, urinary titin levels were significantly higher in the sarcopenia subgroup (5.78 vs. 2.79 pmol/mgCr, *p* = 0.008), and multivariate analyses identified urinary titin as an independent predictor of sarcopenia (odds ratio 13.4, *p* = 0.028). The receiver operating characteristic (ROC) analysis demonstrated fair diagnostic performance (area under the curve [AUC] 0.729), with an optimal cutoff value of 3.676 pmol/mgCr. Urinary titin may serve as a useful non-invasive diagnostic biomarker for sarcopenia in patients with unresectable digestive malignancies, particularly in males. These findings suggest that sex-specific approaches are required for sarcopenia assessment with urinary titin.

## 1. Introduction

Sarcopenia is a condition characterized by declining skeletal muscle mass and function [1]. Epidemiological studies in Japan have reported a prevalence of sarcopenia of approximately 10–20% among older adults [2,3]. The clinical importance of sarcopenia is widely recognized, and some studies have demonstrated effects in suppressing muscle loss [4,5]. However, these findings remain at the investigational stage, and no standard treatment has yet been established. Its presence has also been associated with poor clinical outcomes in digestive malignancies such as pancreatic cancer and colorectal cancer [6,7]. Furthermore, it has been reported that sarcopenia potentially makes chemotherapy more toxic [8,9]. Given this impact, an accurate diagnostic method is urgently needed to improve the prognoses of malignancies. Various diagnostic modalities are utilized for sarcopenia, including gait speed, handgrip strength, bioelectrical impedance analysis (BIA), and computed tomography (CT)-based measurement of skeletal muscle mass. Although gait speed and handgrip strength are non-invasive methods, they heavily depend on patient cooperation, and symptoms related to primary malignancies—such as pain or mobility limitations—may affect the measurement outcomes. BIA is also a simple and non-invasive tool; however, its accuracy can be significantly impacted by device variability, hydration status, and the presence of pleural or ascitic effusions [10]. CT-based evaluation provides an objective quantification of muscle mass; however, manual measurement is prone to errors, and concerns regarding radiation exposure and high costs still remain an issue. Therefore, there is an increasing need for a simple, non-invasive, and objective biomarker for sarcopenia assessment, particularly in patients with cancer.

Furthermore, according to previous studies, sarcopenia manifests differently between male and female patients. Specifically, the absolute reduction in skeletal muscle mass has been reported to be greater in males than in females [11]. Moreover, the association between muscle mass and long-term survival was statistically significant in the former group but not in the latter group [12]. These sex differences can be explained by underlying hormonal or metabolic factors [13,14]. These findings underscore the need for sex-specific considerations in the diagnosis and management of sarcopenia.

Urinary titin has recently emerged as a promising biomarker of muscle damage. Titin, a giant protein that constitutes a sarcomere—the smallest unit of striated muscle—functions as a molecular spring responsible for the development of retractive force during stretching of a nonactivated muscle [15]. Recently, it has been suggested that urinary titin can be an indicator of sarcopenia in preoperative patients with digestive malignancies [16]. In addition, in patients with type 2 diabetes, higher urinary titin concentrations are associated with the diagnosis of sarcopenia in male patients, whereas no such association was observed in female patients. This indicates a sex difference in the association between urinary titin concentrations and sarcopenia diagnosis [17]. However, to the best of our knowledge, there have been no reports on this association in patients with unresectable gastrointestinal cancer. In addition, its utility in sarcopenia diagnostics, particularly with respect to sex differences, remains unclear.

In this study, we aimed to investigate whether urinary titin could be an effective diagnostic marker for sarcopenia in patients with unresectable gastrointestinal malignancies, with a particular focus on possible sex differences.

## 2. Results

### 2.1. Baseline Characteristics of Study Participants

**Figure 1:**
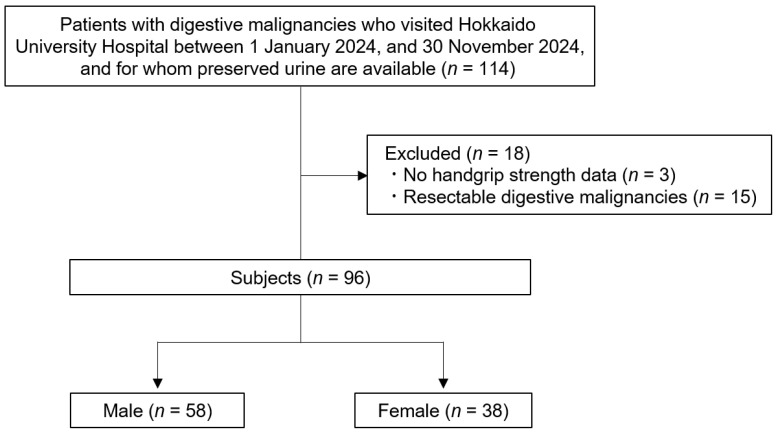
Flowchart of the participant inclusion process.

The flowchart of this study is shown in Figure 1. Of 114 patients with digestive malignancies, 18 ineligible ones were excluded, resulting in the enrolment of 96 patients, whose clinical characteristics are shown in Table 1. There were 58 male and 38 female patients with a median age of 70 (43–85) years. The most common tumor type was pancreatic cancer (38.5%), followed by biliary tract cancer (22.6%). The details of chemotherapy regimens administered to each patient group are summarized in Supplementary Table S1. A total of 34 (35.4%) patients were diagnosed with sarcopenia, including 15 males (25.9%) and 19 females (50.0%). The median urinary titin level in the whole sample was 3.67 (pmol/mgCr).

Table 2 shows the characteristics of patients with or without sarcopenia. Male patients with sarcopenia were significantly older (*p* = 0.001) and had poorer performance status (*p* < 0.001) than female ones. Body mass index (*p* = 0.002), albumin (Alb) (*p* = 0.026), cholinesterase (*p* = 0.035), and geriatric nutritional risk index (*p* = 0.001) were significantly lower among patients with sarcopenia. Urinary titin levels were also significantly higher among patients with sarcopenia (2.79 [95% CI: 0.54–23.8] vs. 5.78 [1.21–96.6], *p* = 0.008). In contrast, among female patients, only the body fat percentage was significantly lower among patients with sarcopenia (*p* = 0.012), with no other observed significant differences.

### 2.2. Correlations Between Urinary Titin Levels and Age, Muscle Indicators, and Blood Chemical Parameters

**Figure 2:**
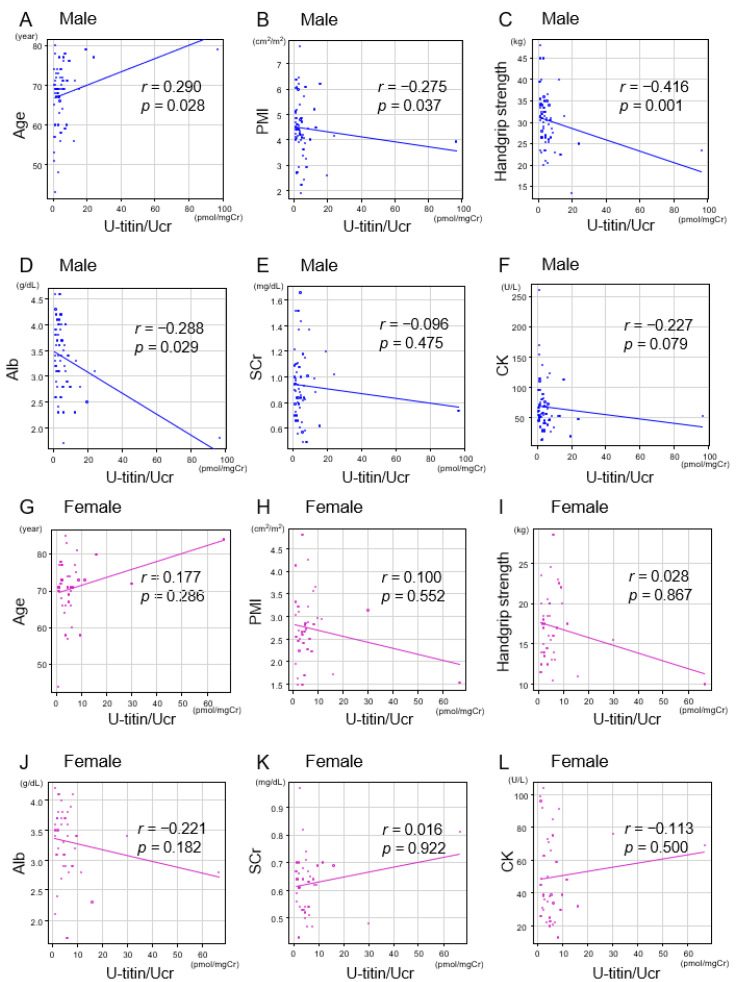
Correlations between urinary titin levels and age, muscle indicators, and blood chemical parameters in male and female patients. Correlations between urinary titin and various clinical parameters are shown. (**A**–**F**) Male patients: (**A**) age, (**B**) PMI, (**C**) handgrip strength, (**D**) Alb, (**E**) SCr, and (**F**) CK. (**G**–**L**) Female patients: (**G**) age, (**H**) PMI, (**I**) handgrip strength, (**J**) Alb, (**K**) SCr, and (**L**) CK. Each scatter plot displays the Spearman’s rank correlation coefficient (r) and *p*-value. Alb, albumin; CK, creatine kinase; PMI, psoas muscle index; SCr, serum creatinine; Ucr, urinary creatinine; U-titin, urinary titin.

Among male patients, significant correlations were observed between urinary titin (U-titin)/urinary creatinine (Ucr) and several clinical parameters. Age was the only parameter with a significant positive correlation with U-titin/Ucr (r = 0.290, *p* = 0.028, Figure 2A). U-titin/Ucr had significant negative correlations with the psoas muscle index (PMI) (r = −0.275, *p* = 0.037), handgrip strength (r = −0.416, *p* = 0.001), and Alb (r = −0.288, *p* = 0.029) (Figure 2B–D). No significant correlations were observed between U-titin/Ucr and either serum creatinine (r = −0.096, *p* = 0.475) or creatine kinase (r = −0.277, *p* = 0.079) (Figure 2E–F). Similar trends were detected among female patients, but the correlations were not statistically significant (Figure 2G–L). The raw data are available in Supplementary Table S2.

### 2.3. Comparisons of Urinary Titin Levels With or Without the Diagnosis of Sarcopenia and Its Components

**Figure 3:**
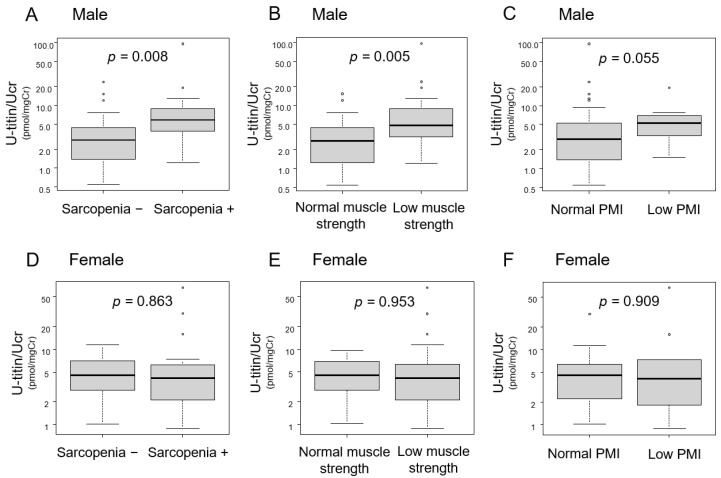
Comparative analysis of urinary titin levels with or without sarcopenia and its components in male and female patients. Urinary titin levels were compared using the Mann–Whitney U test. (**A**–**C**) Male patients: (**A**) with and without sarcopenia, (**B**) with and without low handgrip strength, and (**C**) with and without low PMI. (**D**–**F**) Female patients: (**D**) with and without sarcopenia, (**E**) with and without low handgrip strength, and (**F**) with and without low PMI. *p*-values are indicated in each panel. PMI, psoas muscle index; Ucr, urinary creatinine; U-titin, urinary titin.

Urinary titin levels were comparatively analyzed in the sarcopenia and no-sarcopenia groups stratified by their components (Figure 3). In male patients, urinary titin levels were significantly higher in the subgroups with sarcopenia and low handgrip strength (Figure 3A,B). Although they also tended to be higher in the subgroup with low PMI values, the difference was not statistically significant (Figure 3C). In female patients, urinary titin levels did not differ significantly across all subgroups (Figure 3D–F). Furthermore, to evaluate the interaction effects of sex- and sarcopenia-related factors on urinary titin levels, we performed two-way analyses of variance (ANOVA) (Supplementary Table S3). A significant main effect was observed for the presence of sarcopenia (F = 5.60, *p* = 0.020), as well as low handgrip strength (F = 4.69, *p* = 0.033), but not for low PMI. No significant interaction effects between gender and these factors were identified.

### 2.4. Multivariate Analysis of Factors Contributing to Sarcopenia Diagnosis

We performed the multivariate analysis of factors contributing to the diagnosis of sarcopenia (Table 3). In male patients, urinary titin levels (odds ratio 13.4, 95% confidence interval 1.32–137.0, *p* = 0.028) and Eastern Cooperative Oncology Group performance status (ECOG PS) (odds ratio 34.7, 95% confidence interval 3.06–394.0, *p* = 0.004) were found to be significantly associated with the diagnosis of sarcopenia. In female patients, there were no significant associations with this diagnosis. Additionally, we conducted a multivariate logistic regression analysis including the presence or absence of pancreatic cancer and chemotherapy history as covariates. This model also yielded results similar to the previous analysis (Supplementary Table S4).

### 2.5. Evaluation of the Diagnostic Performance of Urinary Titin by the ROC Analysis

**Figure 4:**
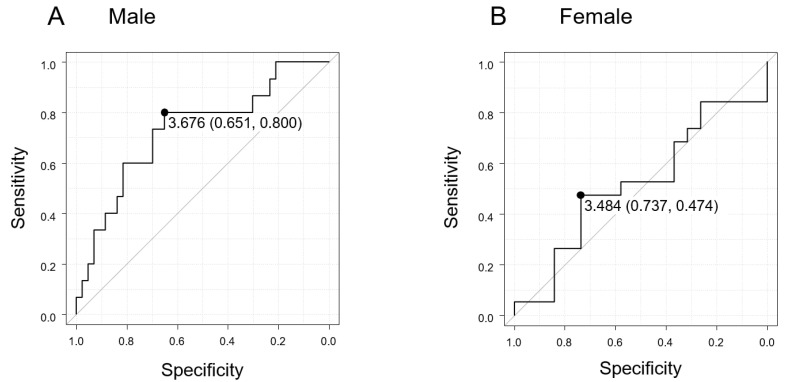
ROC curve demonstrating the diagnostic performance of urinary titin levels for sarcopenia. ROC analyses were performed to determine the optimal cutoff values of U-Titin/Ucr for detecting sarcopenia. (**A**) Male patients: The AUC was 0.729 (95% CI: 0.573–0.884), and the optimal cutoff value was 3.676. (**B**) Female patients: The AUC was 0.518 (95% CI: 0.326–0.710), and the optimal cutoff value composed 3.484. AUC, area under the curve; CI, confidence interval; ROC, receiver operating characteristic; Ucr, urinary creatinine; U-titin, urinary titin.

The receiver operating characteristic (ROC) analysis was performed to obtain the cutoff value of U-titin/Ucr for diagnosing sarcopenia (Figure 4). The area under the ROC curve (AUC) in male patients was 0.729 (95% CI: 0.573–0.884), indicating a good discriminatory ability. The optimal cutoff value determined by the Youden index was 3.676, with a diagnostic accuracy of 72.4%. This accuracy remained consistent regardless of age (Supplementary Table S5). In contrast, in female patients, the AUC was 0.518 (95% CI: 0.326–0.710), with an optimal cutoff value of 3.484 and 42.1% diagnostic accuracy, suggesting limited discriminative ability (Table 4).

## 3. Discussion

In this study, we evaluated the application of urinary titin as a diagnostic biomarker for sarcopenia in patients with unresectable digestive malignancies, focusing on sex, analyzing male and female patients separately. Among male patients, urinary titin levels were significantly higher in those with sarcopenia. Importantly, multivariate logistic regression analysis revealed that urinary titin was independently associated with the diagnosis of sarcopenia in males, even after adjusting for confounding factors such as age, performance status, and nutritional status. These findings strengthen the evidence supporting urinary titin as a robust and independent biomarker for sarcopenia in this population. However, no significant association was observed in female patients. To the best of our knowledge, this study also presents the largest cohort of cancer patients assessed for urinary titin levels to date.

Our findings suggest that urinary titin levels can serve as a useful biomarker for diagnosing sarcopenia in patients with unresectable digestive malignancies, particularly among males. The correlation coefficients between urinary titin and sarcopenia-related parameters were moderate (r ≈ 0.2–0.4), which is consistent with the findings reported by Miyoshi et al. [16], although their study focused on patients with resectable digestive malignancies. In addition, although the AUC derived from the ROC analysis in male patients was below 0.8, the test remains highly valuable given its simplicity and non-invasiveness. The AUC also tended to be higher in males than in females, and the value itself was comparable to that reported by Takiguchi et al. [17]. These results support the clinical utility of urinary titin as a practical biomarker with moderate diagnostic accuracy for assessing sarcopenia in patients with digestive malignancies. Given the poor prognosis and high burden of chemotherapy-related toxicity in patients with unresectable digestive malignancies, early and non-invasive detection of sarcopenia is of significant clinical relevance. The ability to identify at-risk patients using a simple urinary biomarker can facilitate timely interventions such as nutritional support, rehabilitation, and dose modification in chemotherapy.

In this study, we used the PMI rather than the skeletal muscle index to assess muscle mass. PMI can be easily and reliably measured using routine abdominal CT scans without the need for additional image reconstruction or software, making it practical for use in clinical settings. Although PMI does not fully reflect total skeletal muscle mass, it has been reported as a reliable surrogate marker that correlates well with clinical outcomes such as overall survival and treatment tolerance in patients with digestive malignancies [18,19]. Thus, PMI was considered a clinically meaningful indicator in the context of this study.

This study also demonstrated a sex-specific difference in the association between sarcopenia and urinary titin levels among patients with unresectable digestive malignancies. To our knowledge, this is the first study to demonstrate sex differences in the relationship between sarcopenia and urinary titin levels in this category. Takiguchi et al. [17] reported that, in a study involving patients with type 2 diabetes, urinary titin levels correlated negatively with handgrip strength in male patients, whereas no such correlation was observed in female ones, suggesting the existence of a sex difference in the association between urinary titin levels and components of sarcopenia. Furthermore, the optimal cutoff value of urinary titin identified in our study (3.676 pmol/mgCr) was close to that reported by Takiguchi et al. (3.87 pmol/mgCr) [13].

The exact reason for the sex difference in the diagnostic utility of urinary titin levels for sarcopenia is currently unknown. Differences in sex hormone levels, including testosterone and estrogen, are presumed to contribute to sex-specific characteristics of sarcopenia [20]. In addition, female organisms tend to store more fat and preferentially utilize it rather than skeletal muscle as an energy source [14]. In male organisms, muscle protein breakdown is considered to progress more readily due to aging and hormonal changes, and recovery from muscle fatigue has been reported to be slower in them compared to females [21]. These findings suggest that the latter can have greater resistance to muscle damage and a higher muscle preservation capacity. Consequently, it can be hypothesized that urinary titin, a biomarker of muscle injury, may be expressed less in females. These hormonal and metabolic sex differences may account for potential sex-based variations in the kinetics and pathways of titin.

Despite these promising findings, several limitations of this study should be acknowledged. First, this was a single-center retrospective study with a limited sample size, particularly among female patients. Second, we did not evaluate longitudinal changes in urinary titin levels, which can provide an additional insight into disease progression and treatment response. Third, an imbalance in baseline characteristics was observed between groups within the male subgroup. We believe that the influence of this imbalance was eliminated through multivariate analysis. To confirm the findings of this study, future prospective studies with well-balanced patient backgrounds will be required.

## 4. Materials and Methods

### 4.1. Patients and Study Design

In this retrospective study, patients with gastrointestinal, hepatobiliary, or pancreatic malignancies who visited Hokkaido University Hospital between 1 January 2024, and 30 November 2024, were included. The following eligibility criteria were applied: (1) being an adult aged ≥ 18 years, (2) having appropriately stored urine samples, and (3) having access to CT imaging. Patients with missing grip strength data or those with resectable digestive malignancies were excluded. ECOG PS and biochemical parameters were assessed by clinical oncologists at the time of hospitalization for treatment, which also coincided with the collection of urine samples for titin measurement. The presence of metastatic lesions was confirmed using CT. The CT scans used were those performed closest to the date of urine collection for titin measurement, within approximately two months. The study population included patients in a range of clinical situations, including those immediately before chemotherapy initiation and those undergoing ongoing treatment.

### 4.2. Ethics

The study was conducted in accordance with the principles outlined in the Declaration of Helsinki and was approved by the Ethics Committee of Hokkaido University Hospital (Clinical Research Number: IRB 024-0178).

### 4.3. Evaluation of Urinary Titin Levels

Urine samples were stored frozen at −80 °C. Urinary titin levels were measured using an enzyme-linked immunosorbent assay kit (#29501 N-Titin Measurement Kit; Immuno Biological Laboratories, Fujioka, Japan) [22]. To avoid the effects of urine concentration or dilution, the titin N-fragment concentration was corrected by urinary creatinine contents and is shown by the following creatinine ratio: U-titin/Ucr (pmol/mgCr) = urinary titin N-fragment (pmol/L)/urinary creatinine (mg/dL)/10 [23].

### 4.4. Diagnosis of Sarcopenia

The PMI was assessed using CT scanning performed for diagnostic or follow-up purposes at the time point closest to the urinary titin level measurements. The PMI was calculated by manually tracing the cross-sectional area of the right and left psoas muscles at the L3 level and normalizing it by the square of the patient’s height (cm^2^/m^2^) [4,24]. BIA was carried out with a body composition analyzer InBody 770 (InBody Japan Inc., Tokyo, Japan) [25]. Handgrip strength was measured using a Smedley dynamometer. Sarcopenia was diagnosed per the criteria of the Japanese Society of Hepatology, 2nd edition [26]. However, regarding muscle atrophy assessment, PMI values of <3.74 cm^2^/m^2^ in males and less than 2.29 cm^2^/m^2^ in females were used as the cutoff [27].

### 4.5. Statistical Analysis

Statistical analysis was performed using EZR version 1.68 (Saitama Medical Center, Jichi Medical University, Saitama, Japan). Continuous variables were analysed using Student’s *t*-test or Mann–Whitney U test, as appropriate. Categorical variables were evaluated using Fisher’s exact test. Two-way ANOVA was performed to assess the main effects and interaction between sex and sarcopenia-related factors (low PMI, low handgrip strength) on urinary titin levels. The relationship between two variables was assessed using Spearman’s rank correlation coefficient. A multivariate logistic regression analysis was conducted to identify factors associated with the diagnosis of sarcopenia. The multivariate analysis included factors that were suggested to be associated with sarcopenia. To evaluate the discriminative ability of urinary titin for identifying sarcopenia, we performed ROC curve analysis. The AUC was calculated to assess diagnostic performance. The optimal cutoff value was determined based on the Youden index. Statistical significance was set at *p* < 0.05.

## 5. Conclusions

Urinary titin seems to be a useful diagnostic biomarker for sarcopenia in male patients with unresectable digestive malignancies. The sex-specific nature of this association underscores the importance of sex differences in sarcopenia screening and management. To validate these findings and assess the clinical utility of urinary titin levels across diverse populations, prospective multicenter studies with larger sample sizes should be conducted.

## Figures and Tables

**Table 1:** Baseline characteristics.

		Male *n* = 58, 60.4%	Female *n* = 38, 39.6%	All *n* = 96
Age, years		69 (43–80)	72 (44–85)	70 (43–85)
ECOG PS	0	45 (77.6)	20 (52.6)	65 (67.7)
	≥1	13 (22.4)	18 (47.4)	31 (32.3)
Tumor types	Esophageal cancer	7 (12.1)	2 (5.3)	9 (9.4)
	Gastric cancer	8 (13.8)	2 (5.3)	10 (10.4)
	Colorectal cancer	10 (17.2)	5 (13.1)	15 (15.6)
	Pancreatic cancer	16 (27.6)	21 (55.2)	37 (38.5)
	Biliary tract cancer	15 (25.9)	6 (15.8)	21 (22.6)
	Others	2 (3.4)	2 (5.3)	4 (4.3)
Number of metastatic site	0–1	38 (65.5)	25 (65.8)	63 (65.6)
	≥2	20 (34.5)	13 (34.2)	33 (34.4)
History of Chemotherapy ^†^	+	30 (51.7)	17 (47.2)	47 (49.0)
	−	28 (48.3)	19 (52.8)	49 (51.0)
BMI (kg/m^2^)		21.9 (16.2–33.1)	21.3 (14.2–31.9)	21.9 (14.2–33.1)
PMI (cm^2^/m^2^)		4.36 (1.90–7.70)	2.71 (1.49–4.80)	3.89 (1.49–7.70)
Handgrip strength (kg)		30.8 (13.5–48.0)	17.0 (10.0–28.5)	25.3 (10.0–48.0)
Sarcopenia		15 (25.9)	19 (50.0)	34 (35.4)
Percent body fat (%) *		19.8 (2.9–35.4)	25.3 (3.0–44.4)	21.0 (2.9–44.4)
Alb (g/dL)		3.4 (1.7–4.6)	3.4 (1.7–4.2)	3.4 (1.7–4.6)
ChE (IU/L)		200 (44–362)	230 (82–486)	214 (44–486)
SCr (mg/dL)		0.91 (0.49–1.66)	0.64 (0.43–0.97)	0.76 (0.43–1.66)
CK (U/L)		56 (13–260)	39 (13–104)	52 (13–260)
CRP (mg/dL)		0.86 (0.02–17.6)	0.24 (0.02–3.36)	0.47 (0.02–17.6)
eGFRcr (mL/min/1.73 m^2^)		64.2 (35.1–130.7)	70.4 (41.3–103.4)	67.6 (35.1–130.7)
GNRI		45.5 (35.0–65.2)	45.2 (30.9–64.5)	45.4 (30.9–65.2)
U-titin/Ucr (pmol/mgCr)		3.29 (0.54–96.6)	4.47 (0.88–66.6)	3.67 (0.54–96.6)

Data are shown as median (range) values or patient numbers. ^†^: defined as having received chemotherapy within the past six months. *: missing values. Abbreviations: Alb, albumin; BMI, body mass index; ChE, cholinesterase; CK, creatine kinase; CRP, c-reactive protein; ECOG PS, Eastern Cooperative Oncology Group performance status; eGFRcr, creatinine-based estimated glomerular filtration rate; GNRI, geriatric nutritional risk index; PMI, psoas muscle index; SCr, serum creatinine; Ucr, urinary creatinine; U-titin, urinary titin.

**Table 2:** Baseline characteristics with or without sarcopenia.

		Male			Female		
		Sarcopenia−	Sarcopenia+	*p*	Sarcopenia−	Sarcopenia+	*p*
		*n* = 43	*n* = 15		*n* = 19	*n* = 19	
Age, y		69 (43–78)	72 (56–80)	0.001	71 (57–85)	72 (44–84)	0.147
ECOG PS	0	40 (93.0)	5 (33.3)	<0.001	11 (57.9)	9 (47.4)	0.746
	≥1	3 (7.0)	10 (66.7)		8 (42.1)	10 (52.6)	
Tumor types							
	Esophageal cancer	6 (14.0)	1 (6.6)	0.443	0	2 (10.5)	0.427
	Gastric cancer	4 (9.3)	4 (26.7)		0	2 (10.5)	
	Colorectal cancer	6 (14.0)	4 (26.7)		3 (15.8)	2 (10.5)	
	Pancreatic cancer	13 (30.2)	3 (30.0)		11 (57.9)	10 (52.6)	
	Biliary tract cancer	12 (27.9)	3 (30.0)		4 (21.0)	2 (10.5)	
	Others	2 (4.6)	0		1 (5.3)	1 (5.3)	
Number of metastatic site	0–1	26 (60.5)	12 (80.0)	0.218	14 (73.7)	11 (57.9)	0.495
	≥2	17 (39.5)	3 (20.0)		5 (26.3)	8 (42.1)	
History of Chemotherapy ^†^	+	23 (53.5)	7 (46.7)	0.767	11 (57.9)	6 (31.6)	0.191
	−	20 (46.5)	8 (53.3)		8 (42.1)	13 (68.4)	
BMI (kg/m^2^)		22.6 (16.9–33.1)	19.8 (16.2–23.5)	0.002	21.5 (18.2–31.9)	20.7 (14.2–27.6)	0.123
Percent body fat (%) *		20.3 (5.7–32.1)	18.1 (2.9–35.4)	0.333	27.7 (19.1–44.4)	19.7 (3.0–43.6)	0.012
Alb (g/dL)		3.6 (2.3–4.6)	3.2 (1.7–4.2)	0.026	3.4 (2.1–4.2)	3.4 (1.7–4.1)	0.725
ChE (IU/L)		224 (105–362)	158 (44–324)	0.035	234 (82–486)	228 (108–319)	0.286
SCr (mg/dL)		0.89 (0.49–1.52)	1.00 (0.49–1.66)	0.145	0.64 (0.50–0.82)	0.63 (0.43–0.97)	0.511
CK (U/L)		59 (13–260)	51 (19–123)	0.052	39 (13–104)	49 (21–99)	0.579
CRP (mg/dL)		1.04 (0.02–17.6)	0.56 (0.03–11.0)	0.729	0.23 (0.02–3.36)	0.25 (0.02–2.33)	0.977
eGFRcr (mL/min/1.73 m^2^)		68.1 (36.1–130.7)	56.9 (35.1–125.6)	0.104	68.7 (41.3–92.5)	70.8 (43.3–103.4)	0.405
GNRI		47.2 (35.5–65.2)	40.1 (35.0–47.5)	0.001	45.3 (40.2–64.5)	42.8 (30.9–56.3)	0.091
U-titin/Ucr (pmol/mgCr)		2.79 (0.54–23.8)	5.78 (1.21–96.6)	0.008	4.49 (1.01–11.4)	4.15 (0.88–66.6)	0.863

Data are shown as median (range) values or patients’ numbers. ^†^: defined as having received chemotherapy within the past six months. *: missing values. Abbreviations: Alb, albumin; BMI, body mass index; ChE, cholinesterase; CK, creatine kinase; CRP, c-reactive protein; ECOG PS, Eastern Cooperative Oncology Group performance status; eGFRcr, creatinine-based estimated glomerular filtration rate; GNRI, geriatric nutritional risk index; SCr, serum creatinine; Ucr, urinary creatinine; U-titin, urinary titin.

**Table 3:** Multivariate logistic regression analyses for sarcopenia.

		Male		Female	
Factor		Odds Ratio (95% CI)	*p*	Odds Ratio (95% CI)	*p*
Age	≥Median	3.95 (0.55–28.6)	0.174	2.92 (0.76–11.3)	0.119
ECOG PS	≥1	34.7 (3.06–394.0)	0.004	1.63 (0.38–6.90)	0.508
GNRI	<Median	0.14 (0.02–1.19)	0.072	1.03 (0.26–4.04)	0.971
U-titin/Ucr	≥Median	13.4 (1.32–137.0)	0.028	0.64 (0.16–2.65)	0.539

Abbreviations: CI, confidence interval; ECOG PS, Eastern Cooperative Oncology Group performance status; GNRI, geriatric nutritional risk index; Ucr, urinary creatinine; U-titin, urinary titin.

**Table 4:** Diagnostic performance of urinary titin for sarcopenia detection assessed by ROC analysis.

U-titin/Ucr (pmol/mgCr)	Sarcopenia+	Sarcopenia−	Accuracy Rate
Male, *n* = 58	*n* = 15	*n* = 43	72.4%
≥3.676	12	15	
<3.676	3	28	
Female, *n* = 38	*n* = 19	*n* = 19	42.1%
≥3.484	11	14	
<3.484	8	5	

Abbreviations: ROC, receiver operating characteristic; Ucr, urinary creatinine; U-titin, urinary titin.

## Data Availability

All data generated or analyzed during this study are included in this article and the Supplementary Material. Further inquiries can be directed to the corresponding author.

## Supplementary Materials

The supporting information can be downloaded at https://www.mdpi.com/article/10.3390/ijms26146781/s1.

## Abbreviations

The following abbreviations are used in this manuscript:

Alb	albumin
ANOVA	analysis of variance
AUC	area under the curve
BIA	bioelectrical impedance analysis
BMI	body mass index
ChE	cholinesterase
CK	creatine kinase
CRP	c-reactive protein
CT	computed tomography
ECOG PS	Eastern Cooperative Oncology Group performance status
eGFRcr	creatinine-based estimated glomerular filtration rate
GNRI	geriatric nutritional risk index
PMI	psoas muscle index
ROC	receiver operating characteristic
SCr	serum creatinine
Ucr	urinary creatinine
U-titin	urinary titin
