# Supplementary material for: Urinary Titin as a Non-Invasive Biomarker for Sarcopenia Sex Differences in Unresectable Digestive Malignancies: A Retrospective Cohort Study
Source: Int J Mol Sci. 2025 Jul 15;26(14):6781. doi: 10.3390/ijms26146781 (PMC12295692; doi:10.3390/ijms26146781)
Supplement: Supplementary file 1 [file ijms-26-06781-s001.zip › ijms-3715650-supplementary.pdf]

Supplementary table S1. Number of patients by tumor type and chemotherapy regimen

**Tumor type**	**Regimen**	**Number of patients**
Esophageal cancer	FP + Pembrolizmab	4
	FP	2
	Others	3
Gastric cancer	FOLFOX + Zolbetuximab	3
	FOLFOX or CapeOX or SOX + Nivolmab	3
	SOX + Trastuzumab	2
	FOLFOX or SOX	2
Colorectal cancer	FOLFOX or CapeOX + Bevasizumab	5
	FOLFOX + Panitumumab	3
	FOLFOX	3
	Others	4
Pancreatic cancer	Gemcitabine + nab-paclitaxel	29
	FOLFIRINOX	2
	Nal-IRI+5-FU/LV	2
	Others	4
Biliary tract cancer	Gemcitabine + cisplatin + duruvalumab	10
	Gemcitabine + cisplatin + S-1	4
	Gemcitabine + cisplatin + pembrolizmab	3
	Others	4
Others	Various	4

Abbreviations: CapeOX, capecitabine + oxaliplatin; FOLFOX, 5-FU + leucovorin + oxaliplatin; FP, 5-FU + cisplatin; LV, leucovorin; Nal-IRI, nanoliposomal irinotecan; SOX, S-1 + oxaliplatin.

Supplementary table S2. Dataset of clinical variables for correlation analysis between U-titin/Ucr and clinical parameters

Patient ID	Sex	Age, y	PMI (cm^2/m^2)	Handgrip Strength (kg)	Alb (g/dL)	SCr (mg/dL)	CK (U/L)	U-titin/Ucr (pmol/mgCr)
01	F	74	2.72	13.0	2.9	0.47	181	5.34
02	M	79	2.58	13.5	2.5	1.20	145	19.26
03	M	71	4.07	40.0	3.6	1.52	181	1.42
04	F	70	4.13	23.5	3.6	0.70	232	1.12
05	M	57	6.05	48.0	4.3	0.79	260	1.04
06	M	53	5.12	39.5	4.6	0.90	362	0.54
07	M	73	4.42	32.0	4.1	1.10	322	1.74
08	M	56	5.21	40.0	2.3	0.99	142	12.07
09	M	58	4.71	31.0	4.0	0.86	296	4.42
10	M	69	4.56	30.0	3.4	0.80	184	2.79
11	M	63	4.19	32.5	3.9	0.76	238	3.80
12	M	72	3.62	20.5	1.7	1.18	44	5.47
13	F	69	2.85	24.5	3.1	0.74	255	4.92
14	M	51	4.51	45.0	3.4	1.22	288	0.8
15	F	78	1.63	15.0	2.4	0.43	136	1.82
16	M	68	4.42	20.0	2.3	0.66	105	2.68
17	M	59	3.25	31.0	3.1	0.58	188	7.01
18	M	57	4.22	34.5	3.9	0.94	224	0.76
19	F	77	2.55	12.5	3.4	0.59	251	1.26
20	F	72	3.22	17.0	3.7	0.66	229	1.96
21	M	74	4.09	30.5	3.7	1.44	263	4.43
22	M	69	4.85	23.5	3.1	1.10	158	1.21
23	M	77	4.15	25.0	3.1	1.02	238	23.83
24	F	80	1.73	11.0	2.3	0.69	108	15.92
25	M	60	5.20	32.5	3.9	0.89	253	1.38
26	F	71	3.04	20.0	4.2	0.56	214	1.02
27	M	65	4.71	31.5	3.3	1.09	263	0.88
28	M	71	4.46	27.5	3.4	1.01	186	8.33
29	F	68	2.70	18.5	3.5	0.70	172	2.11
30	M	60	2.94	28.5	2.9	0.49	210	7.72
31	F	44	1.94	11.5	3.5	0.67	244	0.88
32	F	71	2.51	13.0	2.8	0.65	181	6.54
33	F	71	2.66	20.0	3.3	0.52	203	4.44
34	F	66	3.22	28.5	3.8	0.54	234	5.98
35	F	57	2.81	18.5	3.1	0.54	131	4.58
36	F	81	2.84	22.5	4.1	0.62	486	8.34
37	M	72	2.24	25.5	3.4	1.00	144	4.20
38	M	77	5.73	26.0	3.2	0.84	176	5.96
39	M	67	4.26	31.5	4.6	1.08	251	3.60
40	M	67	5.37	35.0	3.6	0.91	200	3.35
41	M	77	4.79	23.5	3.3	0.88	148	3.68
42	M	76	4.01	23.0	2.8	1.37	103	9.37
43	M	73	6.45	29.0	3.1	0.84	143	2.92
44	M	69	2.44	25.5	2.3	0.49	153	5.78
45	M	71	4.49	22.5	3.3	0.88	178	12.90
46	M	79	3.93	23.5	1.8	0.74	72	96.58

47	M	69	5.98	34.0	3.8	1.11	292	2.35
48	F	64	2.65	14.5	3.6	0.53	214	3.48
49	F	71	2.27	10.5	1.7	0.67	125	5.84
50	F	73	2.94	17.5	2.8	0.70	221	11.41
51	F	74	1.70	20.0	2.9	0.50	148	4.49
52	F	66	2.50	17.5	4.1	0.54	319	2.55
53	F	83	2.41	16.0	3.8	0.53	214	4.15
54	F	70	2.87	16.0	3.4	0.63	298	5.98
55	F	77	2.23	17.0	3.9	0.47	230	7.32
56	M	69	6.20	31.5	2.8	0.62	135	15.34
57	F	75	3.56	23.0	2.9	0.64	169	7.92
58	M	60	7.70	33.5	4.1	1.37	274	3.23
59	M	64	4.19	36.5	2.8	0.57	139	5.29
60	M	67	4.59	26.5	2.8	0.70	130	1.79
61	F	73	2.24	13.5	3.2	0.97	228	2.45
62	M	69	4.15	28.0	3.1	1.04	228	1.91
63	F	77	3.10	14.0	3.8	0.61	203	2.31
64	M	43	4.40	35.5	2.9	0.73	132	1.24
65	F	72	3.14	15.5	3.4	0.48	240	29.94
66	F	73	2.46	18.0	3.7	0.61	289	2.06
67	M	69	6.37	33.5	3.2	0.95	298	1.12
68	F	67	2.74	15.5	3.7	0.71	267	5.02
69	M	74	4.02	34.0	3.8	1.00	199	0.77
70	M	69	4.36	36.0	3.6	0.79	229	3.20
71	F	72	1.49	17.5	3.1	0.64	262	1.68
72	F	58	2.96	20.0	3.4	0.69	333	9.65
73	F	85	1.49	20.5	4.0	0.68	329	3.67
74	M	74	2.92	32.5	2.4	0.56	130	2.59
75	M	70	4.13	30.5	3.2	0.70	224	0.76
76	M	72	2.70	31.0	3.7	0.84	193	1.70
77	M	68	6.05	23.0	3.2	0.66	257	1.38
78	M	69	4.86	27.0	4.2	0.84	349	4.29
79	M	78	6.07	34.5	3.8	0.99	327	1.39
80	M	70	5.04	31.5	2.6	0.95	114	0.96
81	F	71	3.32	14.0	2.1	0.64	82	1.01
82	M	80	2.69	26.5	4.2	0.97	324	1.49
83	F	66	2.59	12.5	2.7	0.82	250	3.73
84	F	60	4.26	14.0	3.6	0.51	322	6.12
85	M	70	3.38	35.0	3.7	0.65	201	5.07
86	F	58	4.80	18.5	3.7	0.64	285	3.67
87	F	84	1.53	10.0	2.8	0.81	147	66.63
88	M	56	1.90	21..0	2.6	1.66	188	4.06
89	M	69	3.91	28.5	3.1	0.94	142	2.63
90	M	78	2.94	26.5	4.0	1.15	244	6.89
91	M	76	3.86	34.0	3.6	0.93	165	6.22
92	M	48	6.21	45.0	4.0	1.52	285	2.98
93	M	66	4.00	26.5	4.2	0.61	120	3.89
94	F	73	3.65	22.0	3.2	0.62	270	9.01
95	M	69	6.08	30.5	4.0	0.83	212	6.79
96	M	71	4.06	29.5	3.5	0.82	259	5.80

Abbreviations: Alb, albumin; CK, creatine kinase; F, female; M, male; PMI, psoas muscle index; SCr, serum creatinine; Ucr, urinary creatinine; U-titin, urinary titin.

Supplementary table S3. Results of two-way analysis of variance (ANOVA) assessing the effects of sarcopenia-related factors and sex

Source	Sum Sq	Df	F value	p value
Factor A (Presence of sarcopenia)	801.8	1	5.60	0.02009
Factor B (Sex)	28.1	1	0.20	0.65901
A × B	105.2	1	0.73	0.39375
Residuals	13180.4	92		

Source	Sum Sq	Df	F value	p value
Factor A (Presence of low PMI)	115.8	1	0.77	0.3838
Factor B (Sex)	84.8	1	0.56	0.4559
A × B	137.0	1	0.91	0.3438
Residuals	13914.6	92		

Source	Sum Sq	Df	F value	p value
Factor A (Presence of low handgrip strength)	675.0	1	4.69	0.03299
Factor B (Sex)	9.9	1	0.07	0.79414
A × B	92.0	1	0.64	0.42633
Residuals	13252.3	92		

Abbreviations: CI, confidence interval; Df, degrees of freedom; Sum Sq, sum of squares.

Supplementary table S4. Multivariate logistic regression analysis for sarcopenia including pancreatic cancer and chemotherapy history as additional covariates

		Male		Female	
**Factor**		**Odds ratio (95% CI)**	***p***	**Odds ratio (95% CI)**	***p***
Age	≥Median	5.12 (0.56-47.1)	0.149	3.39 (0.81-14.2)	0.096
ECOG PS	1-3	39.8 (3.23-490.0)	0.004	1.45 (0.32-6.57)	0.630
GNRI	<Median	0.14 (0.02-1.32)	0.087	0.96 (0.23-3.99)	0.950
Pancreatic cancer	+	0.36 (0.03-4.68)	0.440	0.94 (0.22-4.08)	0.939
History of chemotherapy	+	0.41 (0.06-2.89)	0.373	0.29 (0.06-1.30)	0.105
U-titin/Ucr	≥Median	12.3 (1.03-147.0)	0.047	0.90 (0.18-4.42)	0.895

Abbreviations: CI, confidence interval; ECOG PS, Eastern Cooperative Oncology Group perfor-mance status; GNRI, geriatric nutritional risk index; Ucr, urinary creatinine; U-titin, urinary titin.

Supplementary table S5. Age-stratified diagnostic performance of urinary titin for sarcopenia detection in male patients

U-titin/Ucr (pmol/mgCr)	Sarcopenia+	Sarcopenia−	Accuracy rate
All, *n*=58	*n*=15	*n*=43	
≥ 3.676	12	15	72.4%
< 3.676	3	28	
Age ≥ 65, *n*=43	*n*=14	*n*=29	
≥ 3.676	11	9	72.1%
< 3.676	3	20	
Age ≥ 70, *n*=25	*n*=11	*n*=14	
≥ 3.676	10	5	76.0%
< 3.676	1	9	

Abbreviations: ROC, receiver operating characteristic; Ucr, urinary creatinine; U-titin, urinary titin.
